# Genome-wide analysis of lncRNA in wheat (*Triticum aestivum*) and functional characterization of *TalncR9* in response to drought stress

**DOI:** 10.3389/fpls.2025.1647354

**Published:** 2025-09-04

**Authors:** Lianzhe Wang, Yutao Zhu, Mei Zhao, Dongxiao Liu, Chunli Liao, Huamin Zhang, Yixian Gou, Taotao Li

**Affiliations:** Pingdingshan Health Food Collaborative Innovation Center, School of Life Science and Engineering, Henan University of Urban Construction, Pingdingshan, Henan, China

**Keywords:** wheat, lncRNA, drought, functional characterization, gene silencing, overexpression

## Abstract

Long noncoding RNAs (lncRNAs) play essential roles in a variety of biological processes in plants. While many lncRNAs have been identified, their functional roles in wheat (*Triticum aestivum*) remain largely unknown. In this study, we identified 2830 lncRNAs in wheat using RNA-sequencing data derived from drought treatment, among which 323 were found to be significantly responsive to drought stress. GO and KEGG analyses indicated that the target genes were significantly enriched in categories related to binding and catalytic activities, response to various stimuli, plant hormone signal transduction, and other stress resistance pathways. Additionally, we identified 56 TalncRNAs that could potentially serve as target mimics for 38 different miRNAs. A ceRNA network was constructed, which included 19 lncRNA-miRNA-mRNA interactions, comprising 9 lncRNAs, 6 miRNAs, and 14 mRNAs. Silencing *TalncR9* in wheat reduced drought tolerance, decreased soluble sugar and proline levels, and increasing MDA levels. *TalncR9* overexpression in *Arabidopsis* enhanced drought resistance, increasing germination rates and root length under mannitol treatment. *TalncR9* up-regulated drought-related genes (*LEA30*, *DREB2*, etc.) in transgenic line. These results demonstrate *TalncR9*’s role as a positive drought regulator and provide insights for improving wheat resilience.

## 1 Introduction

Wheat (*Triticum aestivum* L.) is one of the most extensively cultivated crops and is crucial for global food security. The wheat sowing process is highly sensitive to multiple environmental stresses, which can drastically impair the crop yield and quality. Among these stresses, drought impacts on seedling growth and significantly limits grain production, causing severe economic losses. To adapt to drought stress, plants have evolved a complex network of pathways involving signal recognition and conduction, and gene regulation and expression (Shinozaki et al., 2003). Previous research on stress-related gene regulation has primarily concentrated on the function of protein-coding genes, including genes encoding transcription factors, kinases, channel proteins, heat shock proteins, transporters, and antioxidant enzymes (Nakashima et al., 2014). Recently, long noncoding RNAs (lncRNAs) have been recognized as key regulators of gene expression and are implicated in numerous biological processes (Cai et al., 2025).

LncRNAs function in various regulatory processes, such as transcriptional activation, RNA alternative splicing, and chromatin modification (Sun et al., 2018; Wu et al., 2020). lncRNAs (e.g., *COOLAIR*) facilitate RNA-directed DNA methylation (RdDM) and chromatin modifications to silence or activate stress-responsive genes (Xu et al., 2021). Chemical modifications (e.g., m^6^A) on lncRNAs govern their stability, subcellular localization, and interaction with RNA-binding proteins (e.g., writers/erasers), enabling recruitment to nuclear stress bodies or modulation of mRNA translation/stability (Cai et al., 2025). Using RNA-sequencing (RNA-seq) technologies and experimental methodologies, numerous lncRNAs have been identified and confirmed demonstrating their vital roles in multiple abiotic stresses responses in plants (Li et al., 2022; Riyazuddin et al., 2023; Sahraei et al., 2024). For instance, an intergenic *lncRNA1* (*CRIR1*) serves as an innovative positive regulator that influences the plant’s response to cold stress by regulating the expression of stress-related genes in cassava (Li et al., 2022). Overexpression of a nucleus-localized lncRNA (*DRIR*) in *Arabidopsis* increases tolerance to drought and salt (Qin et al., 2017). A nucleus-localized lncRNA (*lncRNA973*) modulates the expression of several salt stress-related genes, thereby regulating the response to salt stress in cotton (Zhang et al., 2019). Moreover, a variety of lncRNAs that respond to abiotic stress have been identified in wheat (Shumayla et al., 2017). The expression of *CSD1* in wheat is indirectly regulated by a *lncRNA* that competes with miR398, affecting the plant’s tolerance to cold (Lu et al., 2020). Four lncRNAs have been cloned from wheat and expression analysis has indicated that they might be involved in modulating or suppressing the protein-coding genes that participate in defense against pathogen infection (Zhang et al., 2013). Construction of an lncRNA-mRNA association network demonstrates that lncRNAs may participate in the response to alkali stress (Wei et al., 2022). Research has indicated that lncRNAs are crucial for stress response in plants, making it essential to explore their functional mechanisms for advancing crop innovation.

Although several lncRNAs have been identified and function in many stress responses, research on drought-responsive lncRNAs in wheat is limited. In the present study, we identified 2830 lncRNAs in wheat from drought treatment-derived RNA-sequencing data, and bioinformatic methods were utilized to investigate the lncRNAs’ potential roles in regulating drought responses. The function analyses of *TalncR9* in gene-silenced and overexpressed plants demonstrated its function in response to drought stress. These comprehensive analyses will enhance our understanding of lncRNA roles in wheat under drought conditions.

## 2 Materials and methods

### 2.1 Identification of lncRNAs and RNA-seq data analysis

The transcriptomic data utilized for lncRNA identification were derived from Liu et al. (2015) (DOI: 10.1186/s12870-015-0511-8). This dataset was originally generated to study the transcriptional response of wheat (*Triticum aestivum* cv. TAM 107) to drought stress, heat stress (Hirose et al.), and combined heat-drought stress (Sahraei et al.). The data are publicly available under NCBI SRA accession number SRP045409 (Liu et al., 2015). TopHat and Cufflinks software were used for transcriptome mapping and assembly (Trapnell et al., 2012). The sequences were screened for protein-coding transcripts and other non-coding RNA families using Blastx, Rfam and the UTR databases (http://utrdb.ba.itb.cnr.it/). The assembled transcripts from this method were used to identify lncRNAs based on these criteria: the transcript should be longer than 200 bp and have an open reading frame < 100 bp; the coding potential calculator value should be < −1. Gene expression levels were determined using FPKM value. Heat maps were created with the log2-transformed FPKM values using Sangerbox software.

### 2.2 Target genes prediction of lncRNAs

Potential cis-target genes were identified as those within 100 kb upstream and downstream of the lncRNA (Zhang et al., 2020). Potential trans-target genes were predicted based on sequence complementarity using the LncTar program (http://www.cuilab.cn/lnctar). The wheat genome database was consulted for functional annotations of target genes, which were then analyzed using the Blast2GO and WEGO programs.

### 2.3 MiRNA target mimicry prediction and ceRNA network construction

PsRobot was used to predict 323 lncRNAs which significantly responsive to drought stress targeted by miRNAs (Wu et al., 2012). Pearson’s correlation coefficients were used to analyze the expression of lncRNAs and mRNAs, and ceRNAs were identified using the ceRNA-score principle (Shu et al., 2019). lncRNAs and mRNAs were selected based on having more than three shared miRNAs, with p-value < 0.05, and FDR value < 0.1. The ceRNA network was constructed by selecting pairs exhibiting negative correlations between miRNAs-mRNAs and positive correlations between lncRNA-mRNAs. The ceRNA network was analyzed and visualized using Cytoscape.

### 2.4 Plant materials and treatment

Wheat ‘Chinese Spring’ plants were used in the study. The plants were cultivated in soil within a greenhouse kept at 25 ± 2 °C with a 16-hour day and 8-hour night light cycle. We treated 10-day-old seedlings with 20% (w/v) PEG6000 in 6h to simulate drought stress (Hu et al., 2013). Seedlings cultivated in a normal (non-stress) environment served as the control group. Leaves of the seedlings were collected for RNA isolation and gene expression analysis.

### 2.5 Gene-silenced plant generation and drought stress treatment

Gene-specific primers were designed from the nucleotide sequence (Traes_5DL_7D14C1A38.1) using Primer6 software and subsequently used to amplify the full *TalncR9* sequence from wheat cDNA. Following sequencing, the suitable fragments were attached to the tobacco rattle virus RNA2 (TRV2) vector. The recombinant plasmids were then transferred into *Agrobacterium tumefaciens* strain GV3101. The TRV2 empty vector was used as a negative control and the phytoene desaturase (*TaPDS*) gene was used as a positive control, which produced a photobleached leaf phenotype. The experiment was performed using the whole-plant silencing method with minor modifications (Zhang et al., 2017). Three experimental replicates were inoculated, and each replicate consisted of more than 20 seedlings. Approximately 2 weeks after virus inoculation, when the *PDS*-silenced seedlings had become white, total RNA was isolated from leaves of both the silenced and control plants for gene expression analyses. The list of primer sequences utilized is provided in **Supplementary Table S1**.

In accordance with previously described methods, two subsets of plants were maintained for drought-stress treatment. The control group was cultivated under a normal (non-stress) environment and the other seedlings were planted at 50% field capacity by withholding water during the entire duration of the study (Manmathan et al., 2013). Leaves from seedlings grown under the non-stress and drought treatments were sampled for physiological indicator measurements.

### 2.6 Transgenic plant generation and drought stress tolerance assay

The complete sequence of *TalncR9* was cloned into a pCAMBIA1304 vector to create overexpression construction. The pCAMBIA1304-*TalncR9* vector and empty vector were introduced into the *A.tumefaciens* EHA105 strain for transformation of *Arabidopsis* (ecotype Columbia 0) via the floral dip technique. The identification of transgenic lines was achieved by DNA PCR. Depending on the transgene expression level, two independent homozygous lines of *TalncR9*-Overexpressed plants (L-5 and L-9) of the T_3_ generation were selected for subsequent drought-stress tolerance assays.

To evaluate drought stress tolerance, sterilized seeds from wild type (WT), vector control (VC), and transgenic lines L-5 and L-9 were germinated in a greenhouse on 1/2 MS medium, with and without mannitol concentrations of 150 mM or 300 mM (Ge et al., 2024). Seed germination rates were recorded for a week. Following 10 days of vertical growth, the length of primary roots was measured. Subsequently, seedlings were collected for RNA extraction and physiological indicators measurements.

### 2.7 Physiological indicator measurement

Physiological indicators were measured using a malondialdehyde (MDA), proline assay, soluble sugar content test, and peroxidase (POD) assay kit (Jiancheng, China) in accordance with the manufacturer’s instructions. Each sample was analyzed in three biological replicates.

### 2.8 Quantitative real-time PCR analysis of gene expression

Total RNA was extracted from the samples and reverse-transcribed with the RNAprep Pure Plant Kit and FastKing cDNA Kit (Tiangen, Beijing, China). Gene expression levels were estimated by qRT-PCR assays, which were performed using gene-specific primers (**Supplementary Table S1**) on a CFX real time PCR machine (Bio-Rad, Hercules, CA, USA). The qRT-PCR primers’ specificity and efficiency were confirmed through melting curve analysis and agarose gel electrophoresis, with each primer pair showing a PCR efficiency of 90%–110%. *β-Actin* were used as the internal reference gene for wheat and *Arabidopsis*, respectively. Relative gene expression levels were determined using the 2^−ΔΔCt^ method. All samples were analyzed with three biological replicates and the data were represented as mean ± standard deviation (SD).

### 2.9 Data availability

RNA-seq data of wheat under heat and drought stress conditions, obtained from the NCBI sequence read archive database, were used to identify lncRNAs (https://www.ncbi.nlm.nih.gov/sra, accession number SRP045409).

## 3 Results

### 3.1 Drought-responsive lncRNAs detected by SRA data

**Figure 1:**
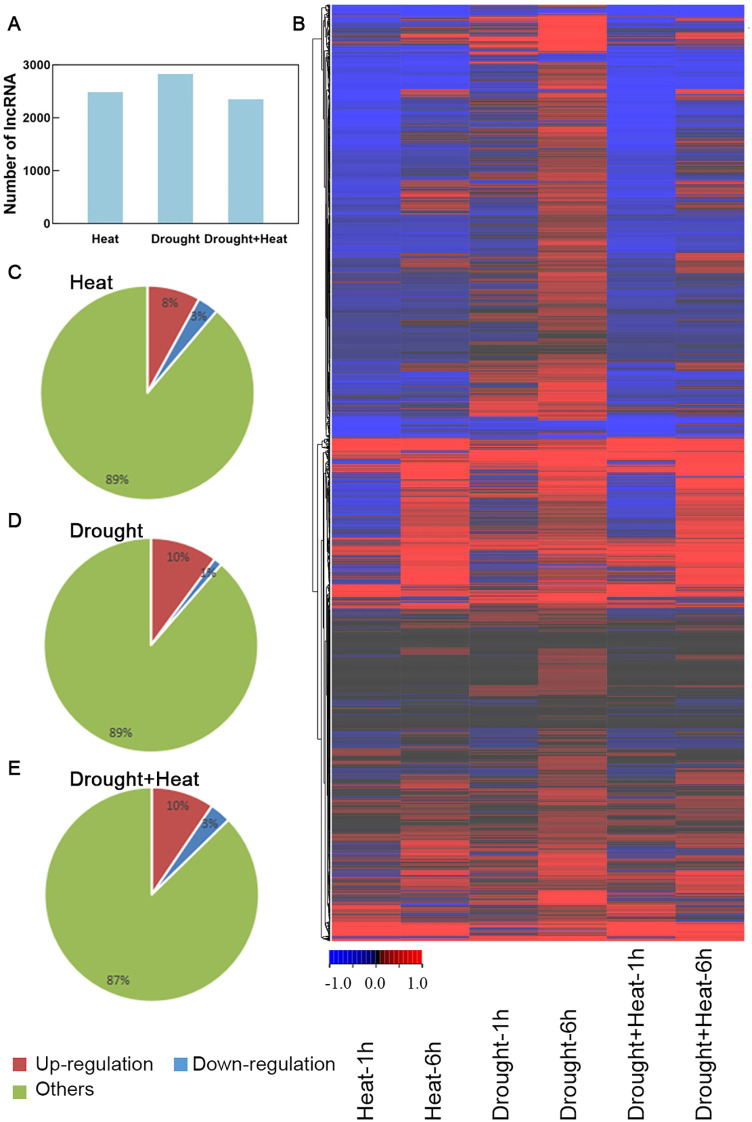
Drought-responsive lncRNAs detected and expression profiles analyses. **(A)** Number of lncRNA detected in SRA data. **(B)** Heat map of drought-responsive lncRNAs in different stresses. **(C–E)** The proportion of up-regulated and down-regulated genes under heat, drought, drought and heat stress treatments.

After *de novo* data assembly and screening, 2830 lncRNA sequences were identified from a total of 101,245 transcripts within the heat- and drought-treatment SRA data. In addition, 2485, 2830, and 2350 lncRNAs were responsive to heat, drought, and heat plus drought stress, respectively (**Figure 1A**). And the 2830 drought-responsive lncRNAs also included 2485 heat-responsive lncRNAs and 2350 drought-heat-responsive lncRNAs. The expression profiles of lncRNAs were analyzed using RNA-seq data. (**Figure 1B**; **Supplementary Table S2**). As the expression levels of a higher number of lncRNAs were significantly changed after 6h of heat or drought treatment than after 1h of the same treatments, the subsequent analyses were based on 6h of stress treatments. The expression levels of 8.0%, 10.1%, and 9.6% of the lncRNAs were significantly up-regulated by heat, drought, and drought plus heat stresses, respectively (value > 1). In contrast, the expression levels of 3.2%, 1.3%, and 3.2% of the lncRNAs were significantly down-regulated by the same stresses, respectively (value < −1) (**Figures 1C–E**). Collectively, 323 significant drought-responsive lncRNAs (value > 1 or < −1) were identified in wheat for further study (**Supplementary Table S3**).

### 3.2 Target genes prediction and GO and KEGG analyses

**Figure 2:**
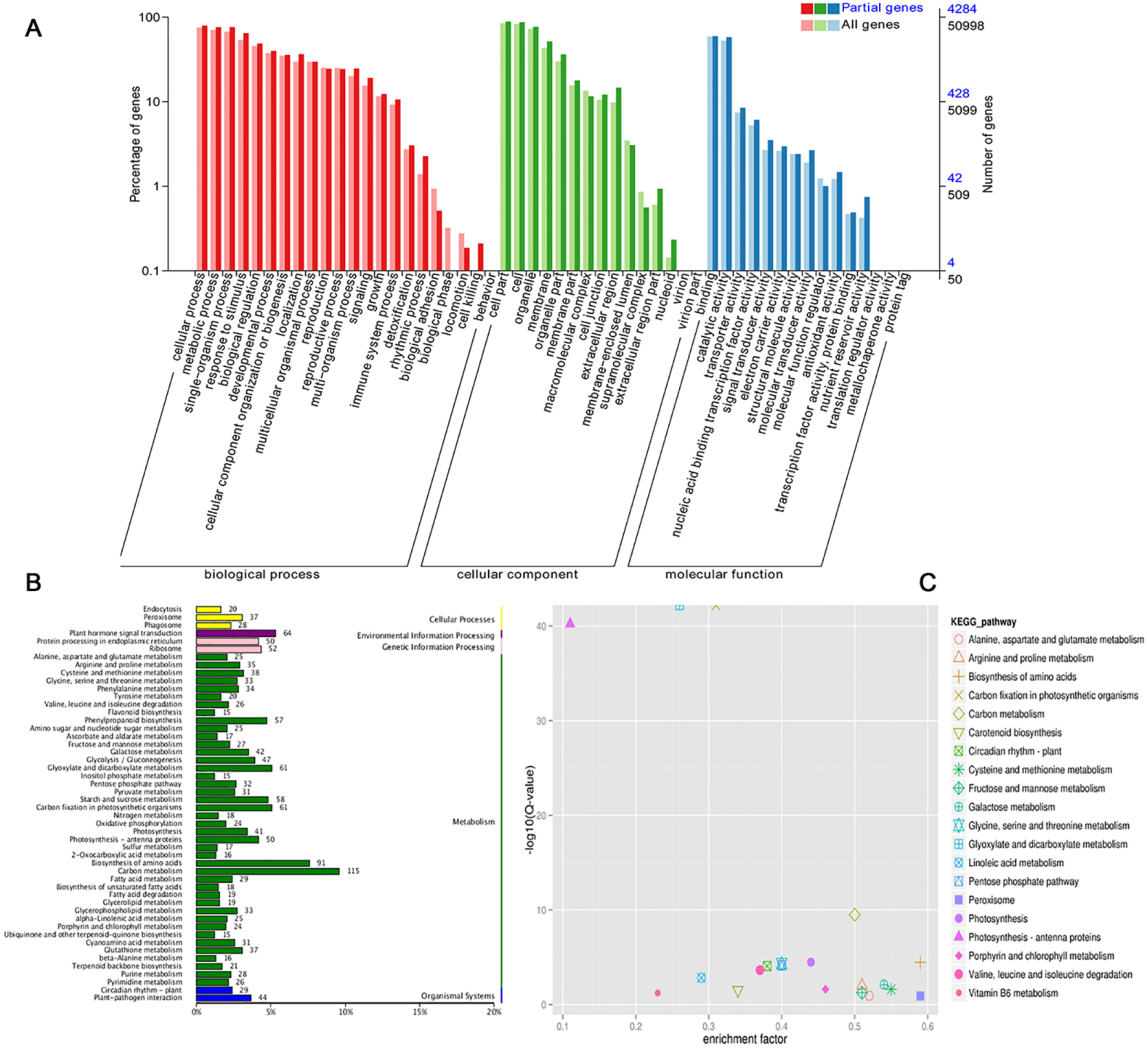
GO and KEGG analyses for predicted target genes. **(A)** Go terms for target genes. **(B)** KEGG classification for target genes. **(C)** KEGG enrichment for target genes.

LncRNA can regulate the expression of target genes by binding to DNA, RNA and protein, and its regulatory modes can be divided into cis-regulation and trans-regulation (Font-Cunill et al., 2018). A total of 6970 target genes were predicted for 323 significant drought-responsive lncRNAs, including 2532 cis-target and 4438 trans-target genes. To identify biological functions, the predicted target genes were organized into GO term classifications. (**Figure 2A**). Consequently, 4284 target genes were classified into the three primary GO categories: biological process (BP), cellular component (CC), and molecular function (MF). In the BP category, the dominant categories are cellular process, metabolic process, and response to stimulus. The most highly represented GO terms in the CC category were cell part, cell and organelle. For the MF category, binding and catalytic activity were the most abundant GO terms. The target genes were also identified to be enriched in additional stress-related terms, including response to jasmonic acid, response to karrikin, response to high light intensity and divalent metal ion transport. These annotations implied that the predicted target genes of drought-responsive lncRNAs are involved in extensive metabolic activities, especially in stress responses.

A metabolic pathway enrichment analysis was performed using the KEGG database to explore the target genes of drought-responsive lncRNAs. We identified 20 significantly enriched KEGG pathways (**Figures 2B, C**). The top three pathways included carbon metabolism (115 target genes), biosynthesis of amino acids (91 target genes), and plant hormone signal transduction (64 target genes). Among these, the pathways most enriched were peroxisome and the metabolism of cysteine and methionine, which are vital for drought response. Peroxisomes help to detoxify reactive oxygen species (ROS), which accumulate during drought stress and can cause oxidative damage (del Río et al., 2002). Cysteine and methionine are involved in the synthesis of antioxidants and redox-active compounds, which are involved in drought stress by regulation of redox state (Reest et al., 2018). These KEGG annotations served as a valuable tool for exploring pathways linked to drought resistance in wheat.

### 3.3 Functional prediction of lncRNAs acting as miRNA target mimics

While some lncRNAs may be degraded by miRNAs (Borah et al., 2018), in the context of target mimicry, lncRNAs competitively bind miRNAs to modulate their activity toward target mRNAs. The psRobot software was utilized to predict possible lncRNA-miRNA target mimics. The results revealed that 56 lncRNAs exhibiting differential expression could act as target mimics for 38 unique miRNAs (see **Supplementary Table S4**). The majority of lncRNAs were associated with a single miRNA, whereas certain miRNAs influenced multiple lncRNAs. For example, tae-miR399, tae-miR9778 and tae-miR9780 targeted four lncRNAs, respectively. Consequently, lncRNAs could substantially influence miRNA activity.

### 3.4 Construction of ceRNA networks related to drought resistance

**Figure 3:**
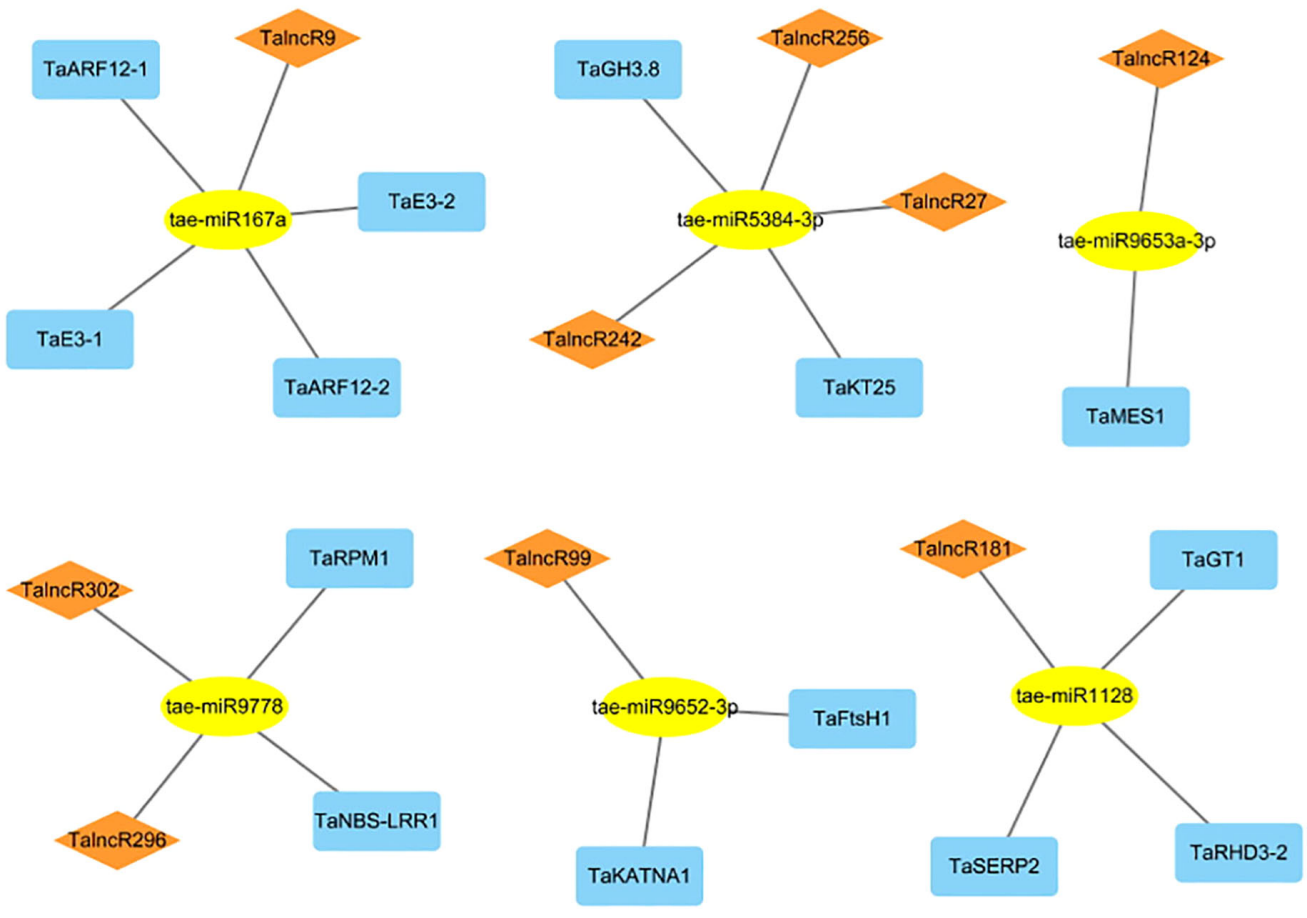
The ceRNA network involving lncRNAs in drought stress response of wheat. Rhomboid, ellipse and rectangle nodes represent lncRNAs, miRNAs and mRNAs, respectively.

A network involving lncRNA, miRNA, and mRNA was constructed in wheat by analysis of 323 lncRNAs which significantly responsive to drought stress. Using the identified mRNAs associated with drought response, we created a network comprising 19 lncRNA-miRNA-mRNA, including 9 lncRNAs, 6 miRNAs, and 14 mRNAs. (**Figure 3**; **Supplementary Table S5**). In this network, 2 target genes (*TaRPM1* and *TaNBS-LRR1*) belong to the NBS-LRR protein family of plants, which are usually responsible for recognizing pathogens and activating downstream immune signaling pathways, could enhance plant disease resistance. These two target genes with 2 lncRNAs (*TalncR296* and *TalncR302*) were targeted by tae-miR9778, involved in ceRNA interactions. Additionally, indole-3-acetic acid-amido synthetase (TaGH3.8) was identified had function in the metabolism of the plant auxin (IAA), and potassium transporter 25 protein (TaKT25) had function in potassium ion transmembrane transporter activity. These 2 target genes along with 3 lncRNAs (*TalncR27*, *TalncR242* and *TalncR256*), were targeted by tae-miR5384-3p. *TalncR9* could targeted 4 genes by tae-miR167a, in which 2 target genes (*TaE3–1* and *TaE3-2*) were identified as RING-type E3 ubiquitin transferase, which played key roles in plant development, stress response, and signal transduction through ubiquitination modification. The other 2 target genes (*TaARF12–1* and *TaARF12-2*) were identified as auxin response factor, which participated in plant development and hormone signal transduction by responding to auxin. *TalncR124*, *TalncR99* and *TalncR181* also involved in ceRNA networks by targeting 1 to 3 target genes, respectively. The ceRNA interactions analysis indicated that lncRNA may play significant roles within ceRNA networks by modulating miRNAs and their downstream target genes in response to drought stress.

### 3.5 Validation of lncRNA expression by qRT-PCR

**Figure 4:**
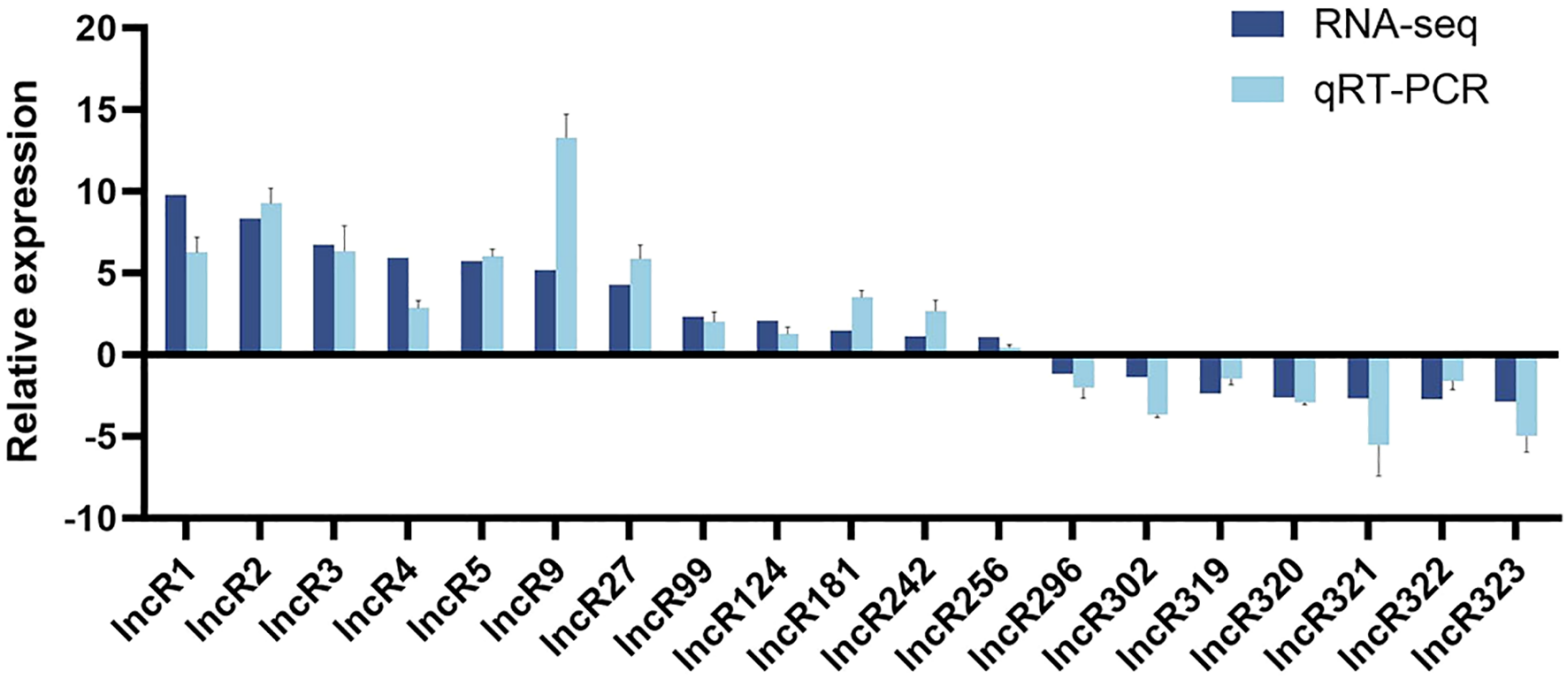
Expression verification of lncRNAs using qRT-PCR and compared with the RNA-seq data. The expression values were log_2_ transformed.

To confirm the RNA-seq data, the 9 lncRNAs involved in ceRNA networks and another 10 most significant up-regulated and down-regulated lncRNAs in response to drought stress were selected for qRT-PCR analysis. After normalization, the expression patterns of the chosen lncRNAs exhibited a similar trend, in which 12 lncRNAs were both up-regulated, and 7 lncRNAs were both down-regulated in RNA-seq data and qRT-PCR analyses. These outcomes suggest that RNA-seq data serve as a reliable source for revealing the expression profiles of lncRNAs in wheat (**Figure 4**).

### 3.6 Gene silencing of *TalncR9* decreases the drought tolerance of wheat

**Figure 5:**
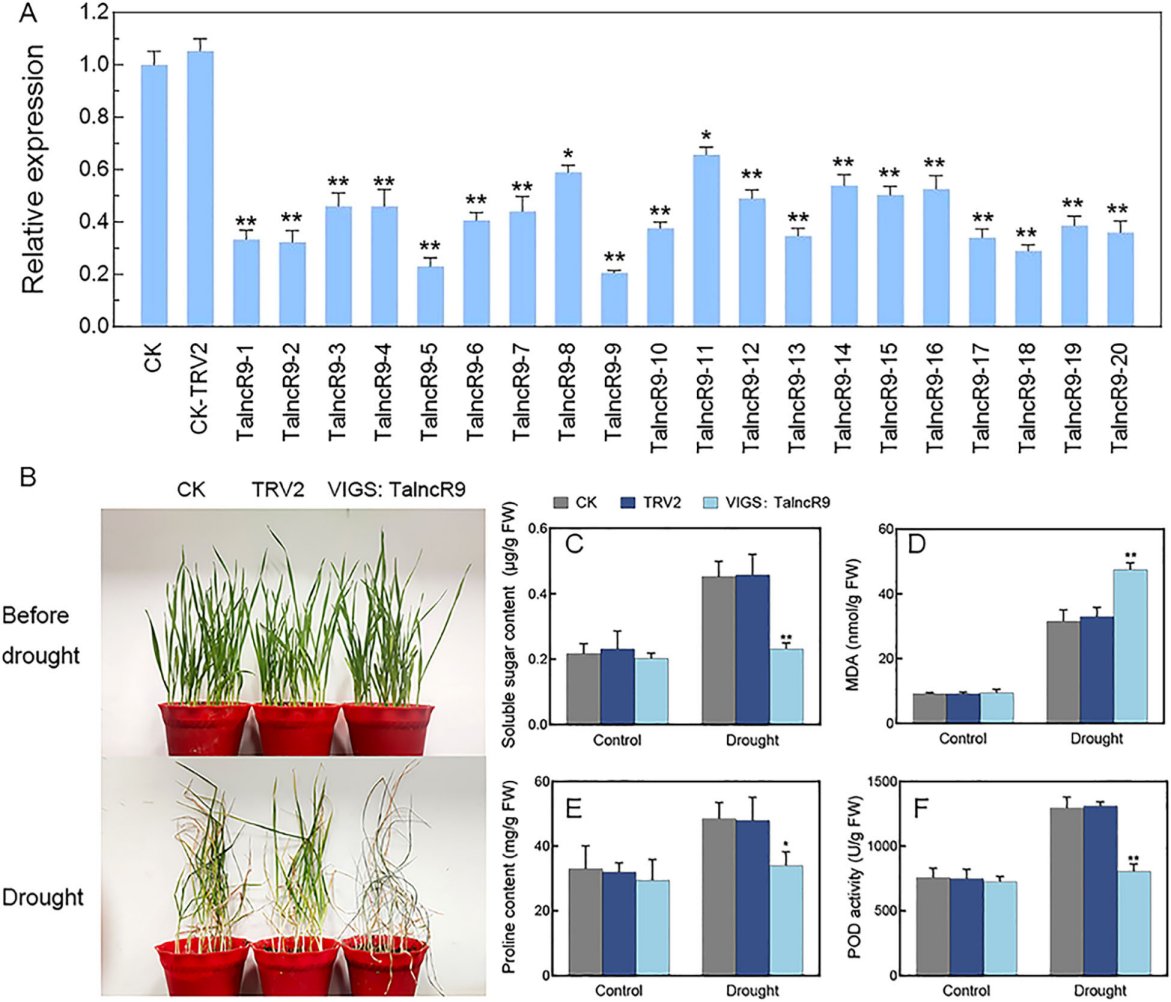
Drought stress responses in *TalncR9*-silenced plants. **(A)** *TalncR9* gene expression in gene-silenced plants. CK, control plants; TRV2, empty vector control plants; *TalncR9*-1-*TalncR9*-20, 20 *TalncR9*-slicened lines. Values represent mean ± SD. **(B)** Phenotypes of the control and gene-silenced groups under drought stress. **(C–F)** Soluble sugar, MDA, proline content and POD activity analyses under drought stress. *TalncR9*-silenced (pooled): Mixed samples from 20 independent silencing lines. Data are means ± SD calculated from three replicates. Significant differences between the gene-silenced plants and control lines are indicated as *P < 0.05; **P < 0.01.

As the qRT-PCR indicated that the expression level of *TalncR9* was increased under drought treatment, and it was involved in ceRNA networks, *TalncR9* may be a potentially significant functional gene implicated in the drought-stress response of wheat. We cloned *TalncR9* for further functional study. The gene-silenced plants of *TalncR9* were constructed by Virus-induced gene silencing (VIGS) technology. The VIGS: *TalncR9* vector was used for inoculation of the wheat seedlings’ two-leaf stage leaves. VIGS: *TaPDS* and VIGS: *TRV2* (CK-TRV2) were served as positive and negative controls, respectively. The photobleached phenotype was observed on the leaves of VIGS: *TaPDS* plants, which indicated that the VIGS system was effective. No differences in the morphology of the VIGS seedlings and the CK (Control Check) seedlings were observed. The qRT-PCR analysis indicated that *TalncR9*’s relative expression level was lower in the majority of VIGS seedlings than in the CK and CK-TRV2 seedlings. (**Figure 5A**). Due to insufficient biomass in individual silenced lines, a pooled sample comprising 20 independent *TalncR9*-silenced lines was used for analysis. After drought treatment, all plants exhibited increased wilting severity. However, the symptoms of VIGS: *TalncR9* plants exhibited more severity compared to the CK and CK-TRV2 plants (**Figure 5B**). Most VIGS: *TalncR9* plants had severely wilted or desiccated leaves, whereas few leaves of the CK and CK-TRV2 seedlings showed yellowing or curling. Physiological indicators—including soluble sugar content, proline content, malondialdehyde (MDA) contents, and peroxidase (POD) activity were measured under drought stress (**Figures 5C–F**). Prior to drought stress, no significant differences were observed in any indicator across the control (CK), empty vector control (CK-TRV2), and *TalncR9*-silenced plants. However, following drought stress: In control groups (CK and CK-TRV2), soluble sugar, proline, and POD activity significantly increased (p<0.05), while MDA content rose moderately. Conversely, in *TalncR9*-silenced plants: Soluble sugar, proline, and POD activity showed either attenuated increases or no significant change, and were significantly lower than controls (p<0.05). Moreover, MDA content exhibited a greater increase, resulting in higher levels than both controls (p<0.01). Collectively, these results demonstrate that silencing *TalncR9* compromises drought tolerance in wheat by impairing osmotic adjustment (via sugar/proline) and antioxidant capacity (via POD), while simultaneously exacerbating membrane lipid peroxidation (via MDA).

### 3.7 Overexpression of *TalncR9* enhances drought stress tolerance in *Arabidopsis*

**Figure 6:**
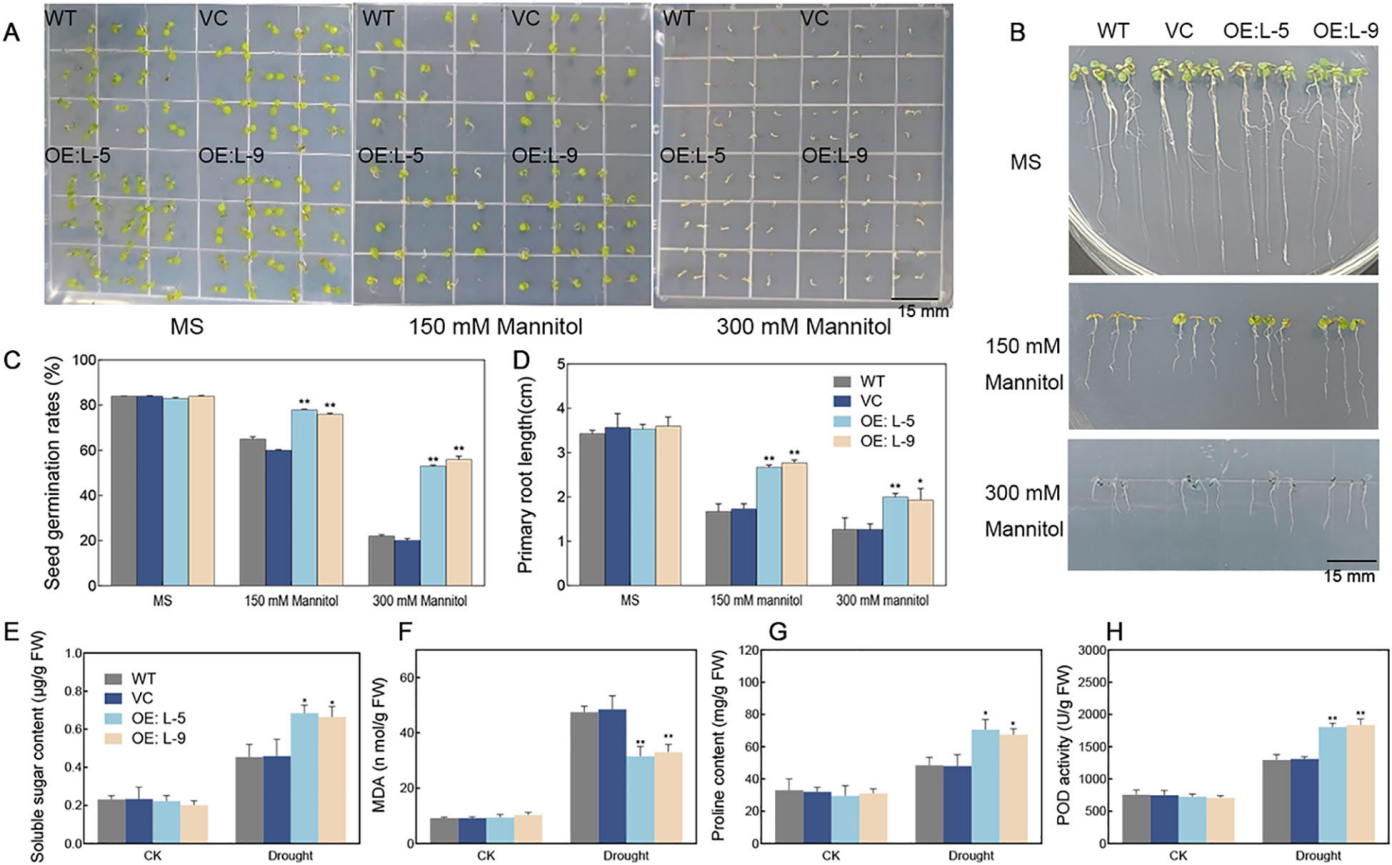
Drought stress responses in *TalncR9*-overexpressed transgenic *Arabidopsis*. **(A)** Photograph of seeds germinated of WT, VC and *TalncR9* transgenic lines L-5 and L-9 on 1/2 MS medium supplemented with or without 150/300 mM mannitol. **(B)** Photograph of primary root length treated with or without 150/300 mM mannitol for 10 days. **(C)** Seed germination rates. **(D)** Primary root length analyses. **(E–H)** Soluble sugar, MDA, proline content and POD activity analyses of different lines under drought stress. Data are means ± SD calculated from at least three replicates. Significant differences between the gene-silenced plants and control lines are indicated as *P < 0.05; **P < 0.01.

To investigate the role of *TalncR9*, we generated transgenic *Arabidopsis* lines overexpressing *TalncR9*. The germination rate and root elongation were examined to investigate the function of *TalncR9* in transgenic plants under drought conditions (**Figure 6**). For the germination rate tests, there were no differences in seed germination rates between transgenic lines and controls (WT, VC) on MS medium under normal conditions. However, the transgenic lines exhibited a greater germination rate compared to the controls on 1/2 MS medium containing 150 mM and 300 mM mannitol. (**Figures 6A, C**). For the root elongation experiment, both control and *TalncR9* transgenic lines grew similarly without treatment, while the transgenic seedlings exhibited greater root lengths compared to the control groups (WT and VC) under mannitol treatments (**Figures 6B, D**).

To examine the physiological processes related to the role of *TalncR9* in drought stress response, the soluble sugar, MDA, proline contents and POD activity were assessed in the transgenic plants and controls. Before drought stress, no significant differences were detected for all analyzed indicators among the WT, VC, and *TalncR9* transgenic lines. After drought stress, the *TalncR9* transgenic plants exhibited lower levels of MDA contents, higher levels of soluble sugar, proline contents, and POD activity compared with the controls (WT and VC) under drought condition (**Figures 6E–H**). The results suggested that overexpression of *TalncR9* improved the drought resistance in transgenic *Arabidopsis*.

### 3.8 Expression analysis of drought-related genes

**Figure 7:**
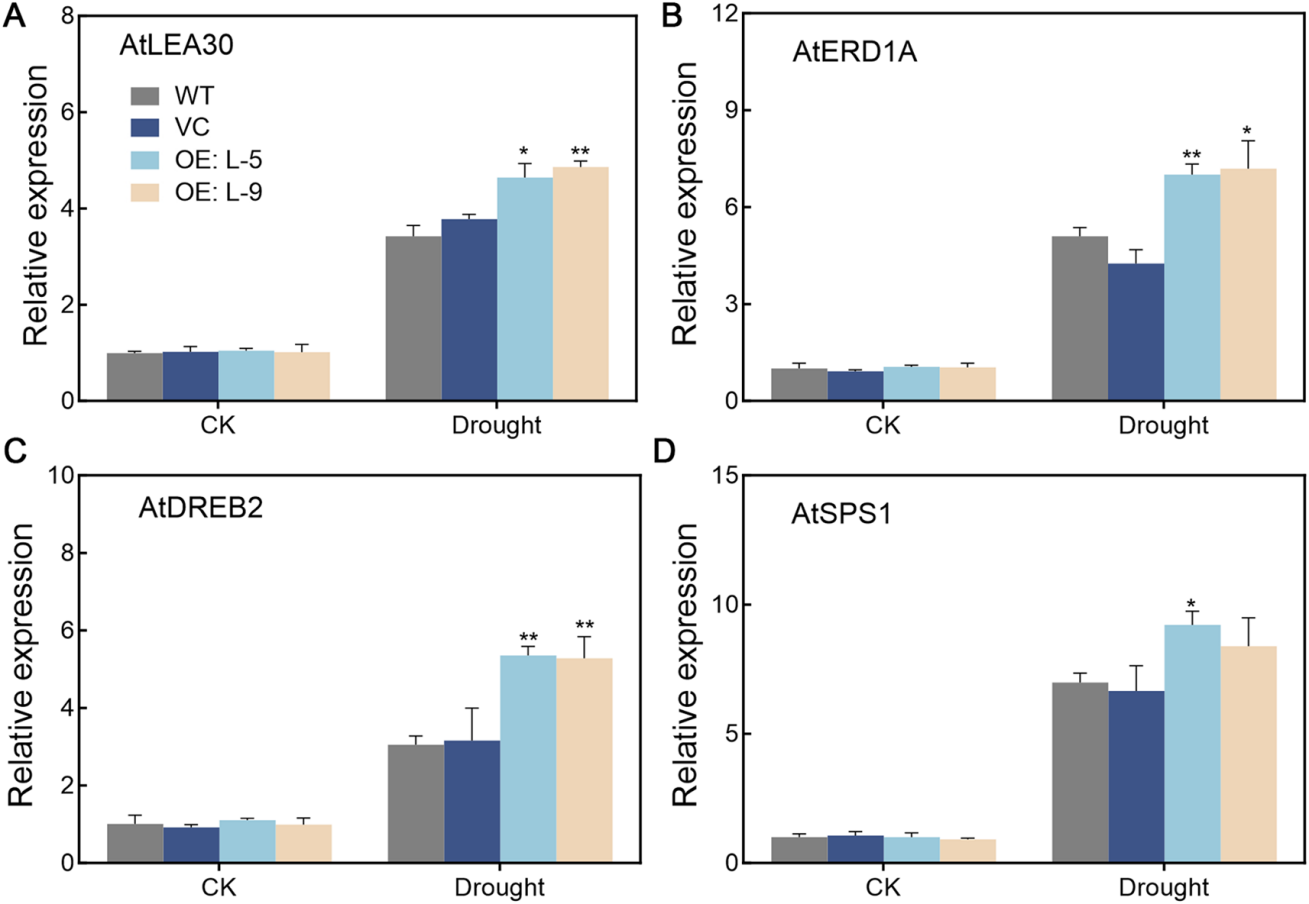
Expression patterns of drought-regulated genes in transgenic plants under normal and drought conditions. **(A)** *AtLEA30* **(B)** *AtERD1A* **(C)** *AtDREB2* **(D)** *AtSPS1*. Data are means ± SD calculated from three replicates. Significant differences between the gene-silenced plants and control lines are indicated as *P < 0.05; **P < 0.01.

The transcript levels of genes involved in drought stress were detected in *TalncR9* transgenic lines and controls (**Figure 7**). Before drought stress, the expression levels showed no significant differences for all analyzed genes among all the lines. Under drought stress, the *AtLEA30*, *AtERD1A*, *AtDREB2* and *AtSPS1* genes were up-regulated, whereas the gene expression levels in the *TalncR9* transgenic plants were higher than in the controls (WT and VC). These findings indicate that *TalncR9* enhances drought tolerance through up-regulation of drought-related genes in transgenic plants.

## 4 Discussion

Drought stress significantly hinders plant development and agricultural yield. So far, numerous genes associated with the response to drought stress have been identified through genetic techniques. However, the lncRNAs that mediate the drought stress responses remain poorly understood (Budak et al., 2015). It was reported that lncRNA plays vital roles in plants’ biological processes, and research on genome-wide investigations of lncRNAs in drought-stressed plants has been conducted across multiple species. For example, a study on sugar beet identified 386 differentially expressed lncRNAs under drought stress conditions (Zou et al., 2023), while another study identified 53 drought-responsive lncRNAs in *Betula platyphylla* (Zhang et al., 2023b). In this study, we systematically identified lncRNAs and analyzed their expression patterns in wheat under drought stress. 2830 lncRNA transcripts were identified from SRA data for drought treatments, of which 323 lncRNAs exhibited significantly increased expression during drought stress (**Figure 1**). These results indicated that lncRNAs may play a role in wheat’s response to drought, aligning with previous research (Shumayla et al., 2017).

Additionally, we identified 6970 transcripts as potential target genes for the 323 key drought-responsive lncRNAs in wheat. GO enrichment and KEGG pathway analyses of these target genes provided insights into their functional roles. GO analysis revealed significant enrichment in metabolic processes, response to stimuli, and binding and catalytic activity (**Figure 2A**). Meanwhile, KEGG analysis highlighted notable enrichment in pathway related to carbon metabolism, amino acids biosynthesis, and plant hormone signal transduction (**Figure 2B**). This suggests that lncRNAs may enhance drought resistance by regulating target genes involved in the metabolism of essential substances. Carbon and nitrogen metabolism supply essential energy and nutrients for plants and are also involved in stress response. As a result, a variety of research have been undertaken to explore how carbon and nitrogen metabolism reacts to both drought and salt stress, particularly focusing on photosynthesis, the metabolism of sucrose and starch, as well as the biosynthesis of amino acids. Overexpression of *ZmSUS1*, a crucial enzyme in carbohydrate processing enhanced drought resistance by managing sucrose metabolism and elevating soluble sugar levels in transgenic maize (Xiao et al., 2024). Amino acid accumulation enhances plant stress resistance through neutralizing reactive oxygen species, modulating pH, and maintaining osmotic balance (Khan et al., 2020). Plant hormones play a crucial role in plant response to stress. External application of MeJA can enhance the drought resistance of grapevines by mitigating oxidative damage and managing carbon and nitrogen metabolism (Zeng et al., 2024). According to our study, the functional processes of target gene enriched pathways play vital roles in the drought response, showing that lncRNA can enhance wheat drought resistance through affecting carbon and nitrogen metabolism and hormone signal transduction.

MiRNAs and lncRNAs play crucial roles in gene regulation, with lncRNAs potentially acting as precursors or target mimics for miRNAs (Borah et al., 2018). 42 DElncRNAs were identified as probable mimics of miRNA targets under drought stress in sugar beet (Zou et al., 2023). 107 DElncRNAs could serve as potential target mimics for 56 miRNAs in sorghum (Zou et al., 2024). According to the current study, 56 DElncRNAs were predicted as target mimics of 38 distinct miRNAs, indicating that lncRNAs are also important in wheat drought stress regulation. Moreover, lncRNAs act as miRNA sponges to compete with endogenous RNA (ceRNA) to mediate mRNA expression and ceRNA networks were widely constructed for plant abiotic stress response (Zhang et al., 2023a). Under drought stress, 673 ceRNA pairs were identified, and 6 were considered hub nodes that enhance peanut drought tolerance (Ren et al., 2022). There were 24 lncRNAs, 7 miRNAs, and 9 mRNAs in the ceRNA networks in sorghum, with the target genes annotated as AAA-ATPases or calcium-dependent protein kinases related to drought stress (Zou et al., 2024). During our study, 19 lncRNA-miRNA mRNA networks were constructed, and some target mRNAs were identified as being involved in plant hormone signal transduction, potassium ion transmembrane transporter activity, E3 ubiquitin transferase, and auxin response factors, suggesting that lncRNAs are involved in miRNA-mediated regulatory processes in wheat under drought stress.

Plant lncRNAs are predicted to have significant functions in response to various environmental stresses. However, functional analysis of lncRNAs in the drought stress response has been poorly investigated in wheat. Wheat is a staple food for many people across the world. Owing to the complex genome and polyploidy, wheat exhibits lower genetic transformation efficiency compared with *Arabidopsis* and rice. In this study, *TalncR9* was silenced in wheat and overexpressed in *Arabidopsis*, and the results indicate that gene silencing of *TalncR9* decreases the drought tolerance of wheat, while overexpression of *TalncR9* increases drought stress tolerance in *Arabidopsis*. Physiological indicators such as soluble sugars, proline and MDA content, which are involved in osmotic balance and cell membrane damage, are useful to assess a plant’s stress tolerance (Kuznetsov and Shevyakova, 1997) (Couée et al., 2006). In previous studies, through phenotypic observation and analysis of physiological indicators (MDA, proline, POD, and superoxide dismutase) of gene-silenced plants under drought stress, silencing of *SLZF57* or *SLB3* decreased drought resistance in tomato (Wang et al., 2020; Gao et al., 2022). Enhanced proline levels and drought resistance in transgenic cassava were achieved through the overexpression of a novel lncRNA (*DIR*) gene (Dong et al., 2022). In this study, *TalncR9*-silenced seedlings exhibited a more severely wilting phenotype, lower soluble sugar and proline contents, lower POD activity, and higher MDA content than those of the control plants, while the *TalncR9*-overexpressed transgenic lines showed the opposite phenotypic and physiological indicators compared to gene-silenced lines (**Figures 5**, **6**). The findings indicated that *TalncR9* might acted as a positive regulator involved in drought stress tolerance in wheat.

The expression patterns of drought stress-related genes are key indicators of plant drought tolerance. The ERD and LEA proteins could improve plant stress tolerance by protecting the structure of cell membrane (Aalto et al., 2012; Mertens et al., 2018). The DREB proteins are transcription factors which could regulate ABA independent genes expression, and improve drought stress resistance in plants (Akhtar et al., 2012). SPS1 protein plays vital roles in the biosynthesis of sucrose, which could offer additional nutrients to plants under drought conditions (Hirose et al., 2014). In this study, the expression levels of drought-related genes increased after drought stress in *TalncR9* transgenic plants, while no variation was observed in plants cultivated on MS medium. The result is consistent with research on overexpression of MdCP37 in apple, as well as Overexpression of *lncRNA77580 in* soybean (Gong et al., 2022; Chen et al., 2023). These findings suggested that *TalncR9* might improve transgenic plants’ drought tolerance by regulating the expression of drought stress-response genes.

## 5 Conclusions

In this study, we identified 323 significant drought-responsive lncRNAs in wheat, and predicted target genes and constructed the ceRNA network. Furthermore, the drought-responsive *TalncR9* was characterized and investigated by gene silencing and overexpression analyses in response to drought stress. Silencing of *TalncR9* leads to decline drought tolerance of wheat, while overexpression of *TalncR9* in *Arabidopsis* can enhance the drought resistance, which may be achieved by regulating stress-response gene expression. Future studies should focus on elucidating the molecular mechanisms of *TalncR9*, including confirmation of its ceRNA network or interaction partners, to facilitate the development of lncRNA-based breeding strategies for drought-resistant wheat. The present results will facilitate further investigation of lncRNAs in wheat to clarify their functions in stress tolerance mechanisms.

## Data Availability

The datasets presented in this study can be found in online repositories. The names of the repository/repositories and accession number(s) can be found in the article/**Supplementary Material**.

## Supplementary material

Supplementary Figure 1 The flowchart of drought-responsive lncRNAs detected in wheat.
