# Supplementary figures and images for: Genome-wide analysis of lncRNA in wheat (Triticum aestivum) and functional characterization of TalncR9 in response to drought stress
Source: Front Plant Sci. 2025 Sep 4;16:1647354. doi: 10.3389/fpls.2025.1647354 (PMC12443737; doi:10.3389/fpls.2025.1647354)

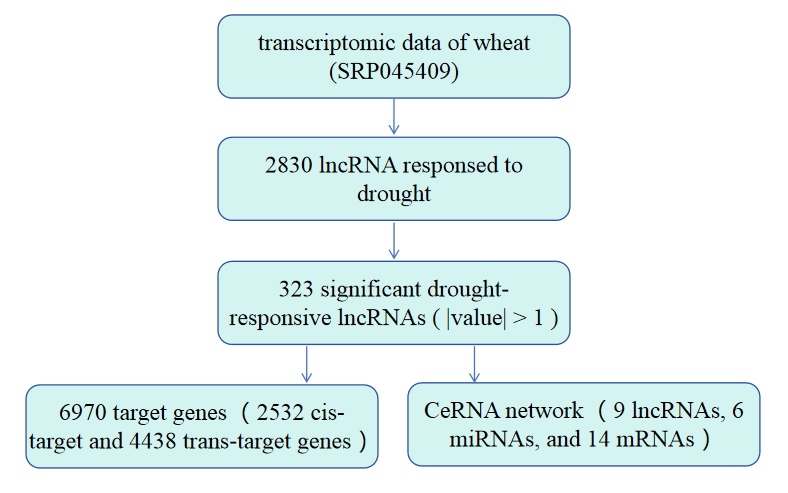

Supplement: Supplementary Figure 1 — The flowchart of drought-responsive lncRNAs detected in wheat. [file Image1.jpeg]
